# Fulton County Medical Society

**Published:** 1871-01

**Authors:** Charles Pinckney


THE GEORGIA

### MEDICAL COMPANION:

A MONTHLY ADVISER.

VOL. I. ATLANTA, GA., JANUARY, 1871. NO. 1.

### PART I.

### Original Communications and Special Selections.

### FULTON COUNTY MEDICAL SOCIETY, ATLANTA, GA.

*Extract from the Minutes of December* 16*th*, 1870. *Reported by* DR. CHARLES PINCKNEY. *Report of cases being in order.*

Dr. Thomas S. Powell reported a case of *Spontaneous Version.* The woman—a negress—had been in labor some fifteen hours. Examination disclosed a complete shoulder presentation. The hand of a dead fœtus was found protruding. In the present advanced stage of the labor, version was supposed to be impossible, and I therefore concluded upon evisceration as the only possible plan of procedure. At this juncture Dr. G. G. Crawford was called in consultation, who fully concurred in the course proposed. A teaspoonful of laudanum was administered to the patient, and we left her, agreeing to return in an hour, with the necessary instruments. We met at the appointed time, and found that though the pains were in no perceptible degree lessened, yet relaxation was so perfect, that we resolved to commence version before resorting to the process of evisceration. Accordingly I forthwith commenced the preliminary manipulations for turning. But now, to our great surprise, the protruded hand was seen receding towards the uterine cavity. Without further interfering with nature, we quietly watched and awaited her work. In a little while the hand was lost to sight, and very soon after the hips presented. A few more pains, and the woman was safely delivered of a dead fœtus.

And now as to the agency which brought about this happy termination of what promised to be a very bad case. In my opinion *laudanum did the work.* Had the drug not been administered, and that, too, in sufficient quantity to meet the indications, it is hardly presumable that relaxation would have occurred in a degree adequate to the occasion, nor would nature, unassisted, have been able to perform her wonderful feat. Without it an operation would certainly have been necessary. The fœtal shoulder and side of the head were greatly contused and swollen, showing the immense efforts made by the womb in its ineffectual attempts at expulsion. The tumefaction of these parts assisted much, no doubt, in the process of rotation.

While spontaneous version, in the case of complete shoulder presentation, is of exceedingly rare occurrence, and in every instance worthy of record, yet I wish to call the attention of the Society more especially to the tincture of opium, with an eye to its relaxing powers over the womb. In view of the facts here presented, I would respectfully urge upon professional brethren the propriety of trying the effects of this drug, in all analagous cases, before appealing to the more hazardous and violent modes of procedure. Such is my practice, except when counter-indicated by peculiar idiosyncracy or special emergency. But while it has been my habit, for years, to give laudanum in order to secure a desired amount of relaxation, by controlling inordinate contractions of the womb, I certainly did not, in the present instance, expect from it such valuable results.

Dr. E. J. Roach reported a case in point, as corroborative of Dr. Powell's idea upon the subject of laudanum. He was called to a woman in tedious labor. The pains had been active for two days and a night, and were now becoming excessive, while the *os uteri* remained undilated, rigid and unyielding. Tr. opii. gtt. 100 were administered with the view of producing relaxation. In a short time the os began to dilate, and six hours after, the labor terminated favorably.

Dr. Pinckney. Was it her first child?

Dr. Roach. It was.

Dr. W. T. Goldsmith reported a case of Cerebro Spinal Meningitis, in which benefit was derived from the use of iodide of potassium. Said the Doctor: In the case of an infant, 18 months old, which occurred in the present month, I completely arrested the convulsive paroxysms by the use of the iodide—mercury being given freely at the same time. The patient lived two weeks, but finally succumbed to the secondary fever, accompanied with trismus, which latter, in a great measure, induced inanition and death. The iodide of potassium is highly recommended by Dr. Ames, of Alabama, in Meningitis, with the statement that it rarely fails to arrest the disease, if sufficient time is had to produce its peculiar effects on the system. In the primary stage, given in large doses, with mercury and counter-irritation to the spinal column, it certainly affords the best means, in my opinion, of combatting this most formidable disease. The secondary fever should be treated with tonics, of which cinchona and its preparations, with steel, are probably the best. Aconite has been recommended in this stage, together with tonics, but I have never given it a trial. I now regret not to have done so in the case reported.

I will also report the case of a negro man, aged 30, who in an affray, received a penetrating wound of the abdomen, three inches long, in the hypo-gastric region, immediately over the spleen. The knife used was six inches in length, and must have entered the cavity to a considerable depth. I inserted my finger its full length, into the wound. The intestines were uninjured, and the spleen seems to have escaped. I found the man writhing in agony. The lips of the wound were united by three sutures, water-dressings applied, and opium given to quiet the patient. He recovered without a single bad symptom, and with but little fever.

Dr. Pinckney said: In pursuance of Dr. Goldsmith's second case, I will report another of the same class, to which I was called on the night of the 21st of August last. S. McG., aged about 22 years, in good health, but of rather dissipated habits, received a knife-wound, an inch and a half long, in the gastric region, near the mesial line. I found him somewhat inebriated, and lying on a bed, at the United States Hotel, in this city, with about six inches of omentum protruding through the external wound. Upon introducing my finger, I discovered that it went directly into the cavity of the stomach itself. The stomach seemed to contain no solid food whatever, and not much liquid. It was nearly empty. The finger, on being withdrawn, smelt strongly of alcohol and coffee. The wound through the gastric coats I judged to be from three quarters of an inch to one inch in length. In view of the alarming nature of the case, a message was sent to my friend Dr. Crawford to come and assist me in its management. Meanwhile I applied myself to the work of replacing the protruded omentum within the abdominal cavity—a task more difficult to accomplish than was anticipated, as the vessels had, by this time, become engorged and the parts somewhat tumefied. When Dr. C. arrived, I had succeeded in returning two-thirds of the escaped omentum, and he very skillfully reduced the rest. The lips of the external wound were then carefully brought together, and secured with adhesive strips, and the patient put under the influence of morphine. The drug was kept up for two or three days, during which time no food was allowed, except a very small amount of milk occasionally. Fortunately for our patient, the stomach-wound, as well as the abdominal parieties, healed by the first intention, and in ten days the man was well.

